# The Role and Mechanism of AMIGO3 in the Formation of Aberrant Neural Circuits After Status Convulsion in Immature Mice

**DOI:** 10.3389/fnmol.2021.748115

**Published:** 2021-09-28

**Authors:** Xue Li, Yanan Pan, Jianxiong Gui, Zhixu Fang, Dishu Huang, Hanyu Luo, Li Cheng, Hengsheng Chen, Xiaojie Song, Li Jiang

**Affiliations:** Chongqing Key Laboratory of Pediatrics, Department of Neurology Children’s Hospital of Chongqing Medical University, National Clinical Research Center for Child Health and Disorders, Ministry of Education Key Laboratory of Child Development and Disorders, Chongqing, China

**Keywords:** status convulsion, aberrant neural circuit, immature mice, AMIGO3, ROCK/RhoA, PI3K/AKT

## Abstract

Leucine rich repeat and immunoglobulin-like domain-containing protein 1 (Lingo-1) has gained considerable interest as a potential therapy for demyelinating diseases since it inhibits axonal regeneration and myelin production. However, the results of clinical trials targeted at Lingo-1 have been unsatisfactory. Amphoterin-induced gene and open reading frame-3 (AMIGO3), which is an analog of Lingo-1, might be an alternative therapeutic target for brain damage. In the present study, we investigated the effects of AMIGO3 on neural circuits in immature mice after status convulsion (SC) induced by kainic acid. The expression of both AMIGO3 and Lingo-1 was significantly increased after SC, with levels maintained to 20 days after SC. Following SC, transmission electron microscopy revealed the impaired microstructure of myelin sheaths and Western blot analysis showed a decrease in myelin basic protein expression, and this damage was alleviated by downregulation of AMIGO3 expression. The ROCK/RhoA signaling pathway was inhibited at 20 days after SC by downregulating AMIGO3 expression. These results indicate that AMIGO3 plays important roles in seizure-induced damage of myelin sheaths as well as axon growth and synaptic plasticity via the ROCK/RhoA signaling pathway.

## Introduction

Seizures in children are more likely to be classified as status convulsion (SC) with adverse neurological outcomes than adults (Reinholdson et al., 2015). Recurrent or prolonged SCs cause formation of abnormal neural circuits, which are manifested as nerve fiber demyelination, mossy fiber sprouting, and structural and functional changes in glutamate receptors, eventually leading to epilepsy, and learning and memory dysfunction (Aldenkamp et al., 2004; Hermann et al., 2007; Schraegle and Titus, 2017). In our previous study, we demonstrated that the myelin sheath, axons and synaptic structure are damaged after SC in immature rats, leading to increased susceptibility to seizures, epilepsy and learning and memory dysfunction in the chronic phase (Song et al., 2018). Learning and memory function have been shown to improve following attenuation of the injury to axons and myelin sheaths (Ye et al., 2013; He et al., 2017; Song et al., 2018). These results suggest that the formation of abnormal neural circuits may be the main cause of learning and memory dysfunction and secondary epilepsy after SC.

Axon growth and myelination in neural circuits are closely related to the differentiation and maturation of oligodendrocytes. Studies have indicated that these processes are regulated by leucine rich repeat and immunoglobulin-like domain-containing protein 1 (Lingo-1) (Mi et al., 2004) and amphoterin-induced gene and open reading frame-3 (AMIGO3) (Foale et al., 2017). In our previous study, we showed that Lingo-1 inhibits axonal regeneration and suppresses myelin production in animal models of SC (Song et al., 2018). However, downregulation of Lingo-1 expression did not reduce the frequency of spontaneous recurrent seizures in the chronic period after SC (Song et al., 2018). Despite preclinical studies suggesting that Lingo-1 downregulation promotes axonal regeneration and remyelination, this promise has not been fulfilled in clinical trials of therapeutics designed to achieve myelin repair (Ledford, 2015; Aktas et al., 2016). It has been suggested that additional mechanisms are involved in the pathogenesis of axonal and myelin injury.

AMIGO3 and Lingo-1 possess an IgC-like domain, which comprises a transmembrane domain and a short cytosolic tail. The only major difference between these proteins is the number of leucine rich repeats (LRRs) (6 for LINGO1, 12 for AMIGO3) in the ectodomain (Jepson et al., 2012; Chen et al., 2006). AMIGO3 is widely expressed in astrocytes, oligodendrocytes and neurons (Kuja-Panula et al., 2003; Ahmed et al., 2005), while Lingo-1 is enriched in axons and oligodendrocytes (Foale et al., 2017). As an analog of Lingo-1, it can be speculated that AMIGO3 exerts similar inhibitory effects on the axonal outgrowth and myelin production via the same signaling pathways (Andrews and Fernandez-Enright, 2015; Sun et al., 2015; Foale et al., 2017). AMIGO3 expression was demonstrated to increase more rapidly than Lingo-1 in an animal model of spinal cord injury (Jepson et al., 2012). The growth of retinal ganglion cells and dorsal root ganglion neurons was promoted after silencing AMIGO3 gene expression in the presence of sphingomyelin *in vitro*, and the RhoA signal pathway was activated after co-transfection of AMIGO3/NGR/p75^*NTR*^ gene in COS7 cells (Ahmed et al., 2013). It is likely that the analogous structures shared by AMIGO3 and Lingo-1 facilitate their interaction with the NgR1 complex, leading to axon growth inhibition (Chen et al., 2006). EGFR/PI3K/AKT has also been implicated as an alternative signal pathway involved in the effect of AMIGO3 on axon growth (Andrews and Fernandez-Enright, 2015; Sun et al., 2015; Foale et al., 2017).

We hypothesized that the failure of this Lingo-1-based therapy may be explained by considering the actions of AMIGO3, which can substitute for Lingo-1, and hence may compensate for therapeutically reduced levels of Lingo-1 function. In this context, regulating AMIGO3 expression will likely have a more rapid benefit by preventing axonal regeneration and demyelination in the early stages after SC, thus providing an alternative therapeutic target for brain damage.

## Materials and Methods

### Adenovirus Vector Construction

Adenovirus vectors encoding a mouse AMIGO3 short hairpin RNA (AV/AMIGO3-shRNA) were constructed and authenticated by the Sangon Biotech Company (Shanghai, China). Furthermore, a green fluorescent protein (GFP) tag was encoded in the adenovirus vector sequence. Three adenovirus vectors were constructed for use in the study: one was used to silence the *AMIGO3* gene (designated AV, sequence: 5′-GCACCACAACCAGACACTTGA-3′), one caused *AMIGO3* gene overexpression (designated OAV), and the third was a negative control adenovirus vector carrying a nonsense gene sequence (designated NC, sequence: 5′-GTTCTCCGAACGTGTCACGTA-3′).

### Animals

Postnatal day 21 [P21] male immature C57/BL6 mouse were provided by the experimental animal center of Chongqing Medical University (China). One hundred and fifty-four immature mice were sacrificed in total. All the mice were housed in a controlled environment (12/12-h light/dark cycle, humidity 50–60%, room temperature 22–24°C). To analyze the sequential changes of Lingo-1 and AMIGO3 in the cortex after SC, eight mice were sacrificed at four time-points (1, 3, 7, and 20 days after SC and at the same age in the control group). To determine the effect of regulating AMIGO3 expression on myelin sheaths, axon growth and synaptic plasticity, we allocated mice to five groups: (1), control group; (2), SC group; (3), SC group treated with AV; (4), SC group treated with OAV; and (5), SC group treated with NC. Six mice were evaluated at 1, 5, and 20 days after SC. All animal procedures were licensed and approved by the Laboratory for Animal Care of Chongqing Medical University.

### Intracerebroventricular Injection and Status Convulsion Model

Five days before inducing SC, the mice were anesthetized via intraperitoneal (i.p.) injection of pentobarbital sodium (7.5 mg/mL, 0.05 mL/10 g) and fixed in a stereotactic frame (RWD Company, Shenzhen, China). A 5-μL Hamilton syringe was placed 0.4 mm posterior and 1 mm lateral to the bregma at a depth of 2 mm relative to the parietal bone and located in the right lateral cerebral ventricle. Each group was injected with 1 μL (0.5 μL/min) virus at the following titers: AV group, 5.21 × 10^10^ PFU/mL; OAV group, 9.95 × 10^10^ PFU/mL; and NC group, 1 × 10^11^PFU/mL. 5 days after the ICV injection, SC was induced in the mice of all SC subgroups by i.p. injection of kainic acid (2 mg/kg, Cayman Chemical Company, Ann Arbor, United States). After 30 min, all mice received a dose of pentobarbital sodium (7.5 mg/mL, 0.05 mL/10 g i.p.) to terminate SC. Mice were excluded from the study due to failure to induce SC failure or death after SC.

### Transmission Electron Microscopy

Mice were anesthetized with pentobarbital sodium (7.5 mg/mL, 0.05 mL/10 g i.p.), and then perfused with 0.9% sodium chloride and a mixture containing 4% paraformaldehyde and 0.5% glutaraldehyde. Hippocampal tissue blocks (1 mm^3^) were dissected and fixed in precooled 2.5% dedicated glutaraldehyde for 2 h at 4°C, and then osmicated in 1% osmium tetroxide (OsO_4_) for 2 h at 4°C. After dehydration in an ascending acetone series, the blocks were infiltrated and embedded with epoxy resin. A single 50-nm section from each epoxy resin block was stained with uranyl acetate for 10–15 min and lead citrate for 1–2 min. Sections were examined under a transmission electron microscope (Tecnai G2 Spirit 120kV, Thermo Fisher Scientific United States) to evaluate the morphological changes in the myelin sheath and synapse. For each section, eight fields of vision were randomly chosen and photographed at magnifications of 6,800× and 30,000×. The diameters of axons and nerve fibers were calculated by measuring the circumference of each by using ImageJ software (NIH Image; U.S. National Institutes of Health, Bethesda, MD, United States). The g-ratios were defined by dividing the axon diameters by the fiber diameters, to provide a reliable index of myelination independent of axonal diameter (Song et al., 2018). The thicknesses of postsynaptic density (PSD) and length of active zone (AZ) were measured for three times in the field of view (30,000× magnification) for each group using ImageJ software. The mean values of PSD thickness and AZ length are used to perform statistical analysis.

### Western Blot Analysis

The cortex of each mouse (*n* = 6 per time-point) was dissected on ice and immediately frozen in liquid nitrogen. Protein samples were extracted using a total protein extraction kit (cat#BB-3101-100T, BestBio, China) and protein concentrations were determined using a protein assay kit (lot# UB276924, Thermo scientific, United States). Samples containing 50 μg of protein and 6 μL Precision Plus Protein Dual Color Standards marker (Cat#1610374, Bio-Rad, California, United States) were separated by 10% polyacrylamide gel electrophoresis (Epizyme, Shanghai, China). Proteins [Lingo-1: 83 kDa; AMIGO3: 55 kDa; myelin basic protein (MBP): 21 kDa; myelin oligodendrocyte glycoprotein (MOG): 28 kDa; neurite outgrowth inhibitor protein A (NogoA): 180 kDa; myelin associated glycoprotein (MAG): 53 kDa; GAPDH 38 kDa; and β-actin: 43 kDa] were transferred to polyvinylidene difluoride (PVDF) membranes (0.2 μm, Bio-Rad, United States) in rapid transfer buffer (Cat. No: WB4600, NCM Biotech, Suzhou, China). The membranes were then blocked for 10 min in blocking buffer (Cat. No: P30500, NCM Biotech, China) before incubation overnight at 4°C with the following primary detection antibodies: rabbit polyclonal anti-Lingo-1 (1:1,000; ab23631, RRID:AB_2135216, Abcam, Cambridge, United States), rat monoclonal anti-AMIGO3 (1 μg/ml; MAB2375, RRID:AB_2226632, Bio-techne, Minneapolis, Minnesota, United States), mouse monoclonal anti-MBP (1:1,000; SMI99, RRID:AB_2314772, Biolegend, California, United States), NogoA (rabbit polyclonal anti-NogoA (1:1,000; ab62024, RRID:AB_956171, Abcam, United States), mouse monoclonal anti-MOG (1:5,000; MAB5680, RRID:AB_1587278, Millipore, Boston, Massachusetts, United States), mouse monoclonal anti-MAG (1:250; ab89780, RRID:AB_2042411, Abcam, United States), mouse monoclonal anti-β-actin (1:1,000; 700068, ZEN BIO, China), or mouse monoclonal anti-GAPDH (1:5,000; 200306-7E4, RRID:AB_2722713, ZEN BIO, Chengdu, China). The membranes were then incubated with the corresponding secondary antibodies for 60 min: goat anti-rabbit IgG (1:5,000; ZB-2301, ZSGB-BIO, Beijing, China), goat anti-rat IgG (1:5,000; ZB-2307, ZSGB-BIO, Beijing, China) or rabbit anti-mouse IgG (1:5,000; 701051, ZEN BIO, Chengdu, China). All proteins are detected on the same blot and sorted out later by targets’ molecular weights. Protein bands were visualized using clarity Western electrochemiluminescence substrate (Cat.1705060, Bio-Rad, United States) and quantified by densitometry using the Syngene imaging system. Protein expression was normalized against β-actin and GAPDH using Image Lab 6.0 software (Bio-Rad, United States).

### Enzyme-Linked Immunosorbent Assay

Commercial ELISA kits (JiangLai Biology, Shanghai, China) were used to determine the concentrations of proteins in the mouse cortex [Lingo-1 (JL49841), AMIGO3 (JL51020), AKT (JL46534), p-AKT (JL20453), RhoA (JL51302), and p-RhoA (JL51309)] according to the manufacturer’s instructions. Briefly, tissue homogenates were added to the ELISA plates and incubated overnight at 4°C. After washing, horseradish peroxidase-conjugated detection antibodies in the commercial kits were added followed by the tetramethylbenzidine substrate solution. Protein concentrations were then quantified by measuring the absorbance of each well at 450 nm using a microplate spectrophotometer (Rayto RT-6100, Shenzhen, China).

### Immunofluorescence Analysis

At 1, 5, and 20 days after SC, mice were anesthetized by i.p. injection of pentobarbital sodium (7.5 mg/mL, 0.05 mL/10 g) and then perfused with 0.9% sodium chloride (50–100 mL) and 4% paraformaldehyde (50 mL) in sequence. After dissection, the brains were then fixed in 4% paraformaldehyde for 24 h, followed by gradient dehydration in sucrose solution (30% followed by 15%). A rotary microtome was used to prepare successive coronal paraffin-embedded sections (3 μm thickness), which were stored at room temperature before incubation overnight at 4°C with the following primary detection antibodies: rabbit polyclonal anti-Lingo-1 (1:200; ab23631, Abcam, United States), rat monoclonal anti-AMIGO3 (25 μg/mL; MAB2375, Bio-techne, Minneapolis, MN, United States). The slides were then incubated with the Alexa Fluor 488 labeled anti-rabbit or Cy3 labeled anti-rat secondary antibodies (Beyotime biotechnology, Shanghai, China, 1:250) for 2 h at room temperature away from light. Images were captured under a fluorescence microscope with the same exposure time (200× magnification).

### Statistical Analysis

Statistical analysis was performed using SPSS 22.0 statistical software. All data were presented as the means ± standard deviation (SD) and were analyzed by one-way or two-way analysis of variance followed by Tukey’s or Sidak’s *post hoc* test. *P* < 0.05 was considered to indicate statistical significance.

## Results

### Dynamic Changes in Amphoterin-Induced Gene and Open Reading Frame-3 and Leucine Rich Repeat and Immunoglobulin-Like Domain-Containing Protein 1 Expression After Status Convulsion

**FIGURE 1:**
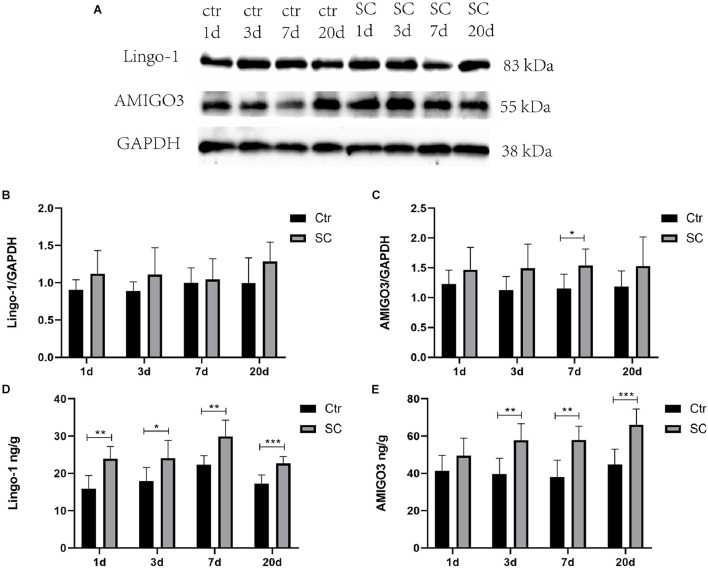
**(A)** Representative western blotting showing the AMIGO3 and Lingo-1 levels in the mouse cortex. **(B)** Lingo-1 levels were normalized to those of GAPDH in the cortex. Values are expressed as Lingo-1/GAPDH ratios based on relative optical densities. **(C)** AMIGO3 levels were normalized to those of GAPDH in the cortex. **(D)** ELISA showing the Lingo-1 levels in the mouse cortex. **(E)** ELISA showing the AMIGO3 levels in the mice cortex. SC: status convulsion. Statistical significance: *p < 0.05, **p < 0.01, ***p < 0.001; *n* = 8.

**FIGURE 2:**
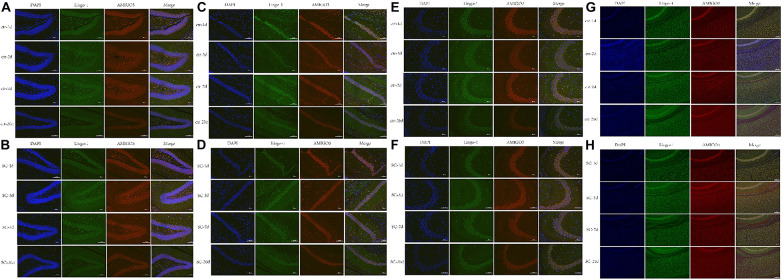
**(A,B)** Representative immunofluorescence showing the AMIGO3 (red) and Lingo-1 (green) expression of control and SC group in the DG zone of hippocampus. **(C,D)** Representative immunofluorescence showing the AMIGO3 (red) and Lingo-1 (green) expression of control and SC group in the CA1 zone of hippocampus. **(E,F)** Representative immunofluorescence showing the AMIGO3 (red) and Lingo-1 (green) expression of control and SC group in the CA3 zone of hippocampus. **(G,H)** Representative immunofluorescence showing the AMIGO3 (red) and Lingo-1 (green) expression of control and SC group in the cortex. Scale bar: 100 μm, SC: status convulsion.

Western blot, ELISA and immunofluorescence analyses were used to detect the changes of the expression of Lingo-1 and AMIGO3 proteins in the cortex. Western blot analysis showed that the expression of both Lingo-1 and AMIGO3 proteins increased in the cortex after SC compared with the levels detected in the control group at the same age (Figures 1A–C). ELISA analysis revealed that Lingo-1 expression in the cortex was significantly increased at days 1, 3, 7, and 20 after SC (*P* < 0.05) (Figure 1D), while significant increases in AMIGO3 expression were detected at days 3, 7, and 20 after SC (*P* < 0.01) (Figure 1E). Furthermore, AMIGO3 expression in the cortex was higher than that of Lingo-1 expression (Figures 1D,E). Paragraph before as shown in Figure 2, immunofluorescence analysis of Lingo-1 (green fluorescence) and AMIGO3 (red fluorescence) revealed similar distributions of the two proteins in the different zones of hippocampus and cortex.

### Amphoterin-Induced Gene and Open Reading Frame-3 Expression After ICV Injection With shRNAs

**FIGURE 3:**
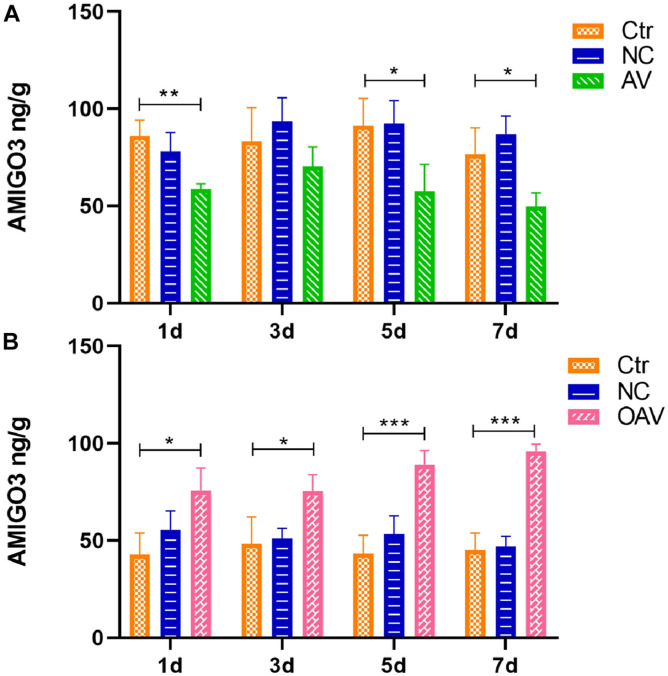
**(A)** ELISA showing the AMIGO3 levels in the mice cortex after intracerebroventricular injection with adenovirus vector to slience the AMIGO3 gene (AV). **(B)** ELISA showing the AMIGO3 levels in the mice cortex after intracerebroventricular injection with adenovirus vector to cause the AMIGO3 gene overexpression (OAV). Statistical significance: *p < 0.05, **p < 0.01; ***p < 0.001; *n* = 4. NC, negative control adenovirus vector carrying a nonsense gene sequence.

The AMIGO3 levels in the cortex after ICV injection with the recombinant adenoviruses was detected by ELISA (Figure 3). No statistical significances were detected between the control and NC groups at different timepoints (Figure 3). Compared with the control group at the same age, AMIGO3 expression in cortex of the AV group was significantly decreased at days 1, 5, and 7 after ICV injection (*P* < 0.05) (Figure 3A). In the OAV group, AMIGO3 expression in the cortex was significantly increased at day 1 after ICV injection, and the levels were maintained to day 7 (*P* < 0.05) (Figure 3B). Based on these observations, SC was induced 5 days after ICV injection with the different recombinant adenoviruses in the subsequent experiments.

### Myelin Sheaths Alterations

#### Ultrastructural Changes in the Myelin Sheath

**FIGURE 4:**
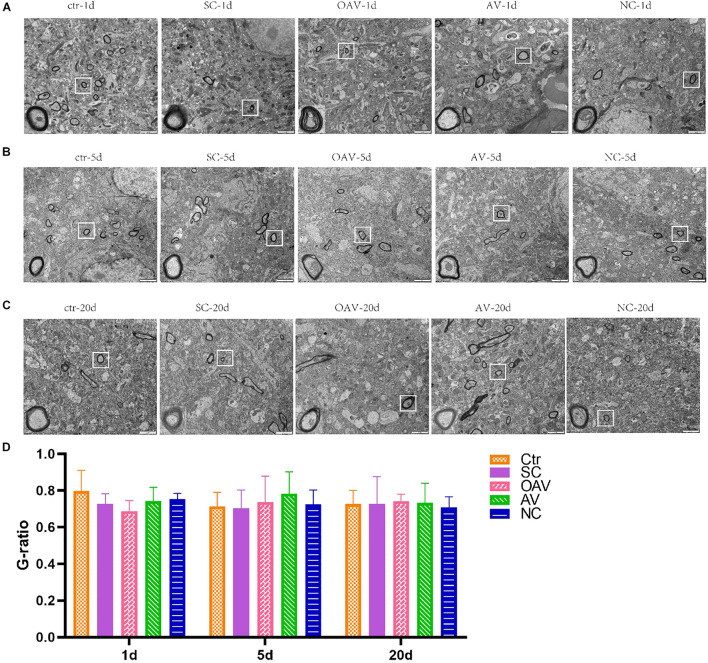
Representative transmission electron microscopy revealing the ultrastructure of myelinated fibers in the immature mice hippocampus (×6,800, scale bar: 2 μm). In each picture, the areas enclosed by a white box were amplified to delineate the myelin sheaths clearly (30000×, scale bar: 500 nm). **(A–C)** The ultrastructure of myelinated fibers at 1, 5, and 20 days after SC. **(D)** Thickness analysis of myelin sheaths based on the quantitation of g-ratios. SC: status convulsion, NC negative control adenovirus vector carrying a nonsense gene sequence, AV adenovirus vectors used to specifically silence the AMIGO3 gene, OAV adenovirus vector for AMIGO3 gene overexpression.

Ultrastructural changes in the myelin sheath in the hippocampus after SC induction were evaluated by TEM. In the control group, the myelin sheath surrounding the neural fibers exhibited regular thickness and a dense structure (Figure 4). After SC induction, the myelin sheath exhibited stratification, vacuolation, collapse, and disruption lasting from 1 to 20 days after SC (Figures 4A–C). The changes in the NC groups were the same as those in the SC groups at 5 and 20 days after SC (Figures 4B,C). The myelin sheaths in the AV groups showed slight damage with little stratification, while those in the OAV groups showed greater damage compared to that in the SC groups at 1 day and 5 days after SC (Figures 4A,B). Myelin sheath thickness in different groups was compared by determined the g-ratios in four randomly selected fields of view. No statistical significances were detected among all the groups at different timepoints (Figure 4D).

#### Changes in Myelin Basic Protein Expression

**FIGURE 5:**
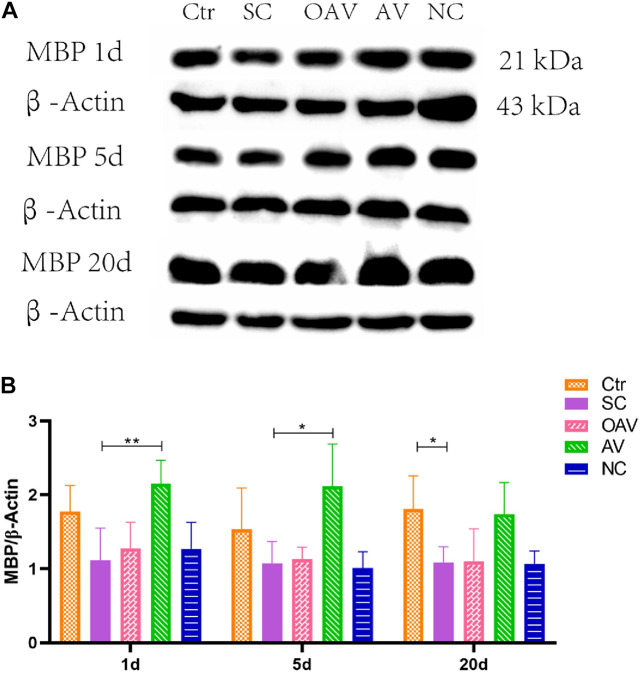
**(A)** Representative western blotting showing the MBP levels of immature mice with different interventions; *n* = 6. **(B)** MBP levels were normalized to those of β-actin in the cortex. Values are expressed as MBP/β-actin ratios based on relative optical densities. All the other groups were compared with SC groups at the same age. *p < 0.05, **p < 0.01. SC: status convulsion. NC negative control adenovirus vector carrying a nonsense gene sequence, AV adenovirus vectors used to specifically silence the AMIGO3 gene, OAV adenovirus vector for AMIGO3 gene overexpression.

Expression of MBP in the cortex was detected by Western blot analysis (Figure 5A). MBP expression was significantly decreased after SC at days 20 after SC (*P* < 0.05) (Figure 5B). Compared with the SC group, MBP expression in the AV group was significantly increased from days1 to 5 after SC (*P* < 0.05) (Figure 5B). There were no significant differences in MBP expression among the SC, OAV and NC groups at any of the time-points.

### Axon Alterations

#### Changes in the Expression of Three Major Myelin-Derived Inhibitors (Neurite Outgrowth Inhibitor Protein A, Myelin Oligodendrocyte Glycoprotein, and Myelin Associated Glycoprotein)

**FIGURE 6:**
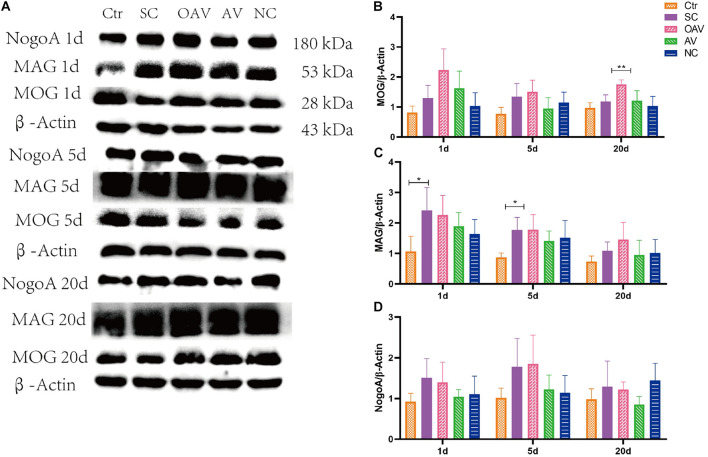
**(A)** Representative western blotting showing the MOG, MAG, and NogoA levels of immature mice with different interventions at different timepoints; *n* = 6. **(B)** MOG levels were normalized to those of β-actin in the cortex. Values are expressed as MOG/β-actin ratios based on relative optical densities. **(C)** MAG levels were normalized to those of β-actin in the cortex. Values are expressed as MAG/β-actin ratios based on relative optical densities. **(D)** NogoA levels were normalized to those of β-actin in the cortex. Values are expressed as NogoA/β-actin ratios based on relative optical densities. All the other groups were compared with SC groups at the same age. *p < 0.05, **p < 0.01. SC: status convulsion. NC negative control adenovirus vector carrying a nonsense gene sequence, AV adenovirus vectors used to specifically silence the AMIGO3 gene, OAV adenovirus vector for AMIGO3 gene overexpression.

Expression of NogoA, MOG, and MAG in the cortex was detected by Western blot analysis (Figure 6A). Compared with the SC group, MOG expression was significantly increased in the OAV group at day 20 (*P* < 0.01) (Figure 6B). Compared with the control group, MAG expressions in the SC group were significantly increased at day 1 and 5 (*P* < 0.05) (Figure 6C). Compared with the SC group, a decreasing trend in MAG expression was observed in the AV groups at day 1 and 5 (Figure 6C). Compared with the SC group, a decreasing trend in NogoA expression was observed in the AV group from days 1 to 20 after SC (Figure 6D). There were no significant differences in the expression levels of NogoA, MOG, and MAG between NC groups and SC groups from days 1 to 20 after SC (Figures 6B–D).

### Synaptic Alterations

**FIGURE 7:**
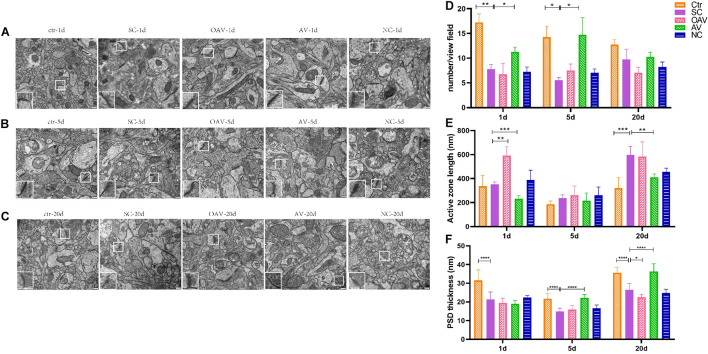
Representative transmission electron microscopy revealing the ultrastructure of synapse in the immature mice hippocampus (30000×, scale: 500 nm). In each picture, the areas enclosed by a white box were amplified to delineate the synapse. **(A–C)** The ultrastructure of synapse at 1, 5, and 20 days after SC. **(D)** The number of synapse with clear structure in each group. **(E)** The length of active zone (AZ) in presynaptic membrane in each group. **(F)** Thickness of postsynaptic density (PSD) in each group. All the other groups were compared with SC groups at the same age. *p < 0.05, **p < 0.01, ***p < 0.001, ****p < 0.0001. SC: status convulsion. NC negative control adenovirus vector carrying a nonsense gene sequence, AV adenovirus vectors used to specifically silence the AMIGO3 gene, OAV adenovirus vector for AMIGO3 gene overexpression.

The functional structures of the presynaptic membrane and postsynaptic membrane were further observed by TEM (Figures 7A–C). In each group, the number of synapses with clearly identifiable structure in the hippocampus was counted in four randomly selected fields of view (20,000× magnification). Compared with the control group, number of synapses was significantly decreased at days 1 and 5 after SC (*P* < 0.05). In contrast, the number of synapses in the AV group was increased at days 1 and 5 compared with the numbers in the SC group (*P* < 0.05) (Figure 7D). The length of the active zone (AZ) of the presynaptic membrane and thickness of postsynaptic density (PSD) in each group was measured and analyzed using ImageJ software (NIH Image; U.S. National Institutes of Health, Bethesda, MD, United States). Compared with the control group, the length of the AZ in presynaptic membrane was increased in the SC group at day 20 (*P* < 0.001). Compared with the SC group, the length of the AZ in presynaptic membrane was increased in the OAV group at day 1, but decreased in the AV group at days 1 and 20 (*P* < 0.05) (Figure 7E). The thickness of PSD significantly decreased from days 1 to 20 after SC when compared with the control group (*P* < 0.0001) (Figure 7F). Compared with the SC group, the thickness of PSD in the AV groups was significantly increased at days 5 and 20 (*P* < 0.0001), while that in the OAV group significantly decreased at day 20 (*P* < 0.05) (Figure 7F).

### Effect of Amphoterin-Induced Gene and Open Reading Frame-3 on ROCK/RhoA and PI3K/AKT Signaling

**FIGURE 8:**
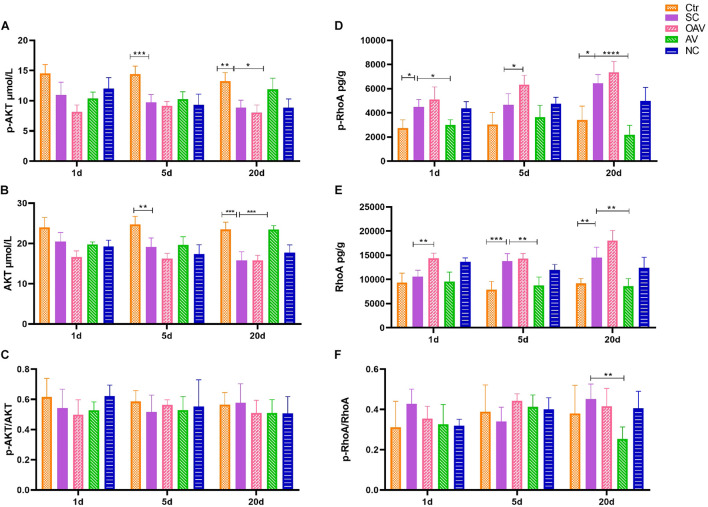
**(A,B)** ELISA showing the p-AKT and AKT levels in the mice cortex. **(C)** ELISA showing p-AKT/AKT ratio in the mice cortex. **(D,E)** ELISA showing the p-RhoA and RhoA levels in the mice cortex. **(F)** ELISA showing p-RhoA/RhoA ratio in the mice cortex. All the other groups were compared with SC groups at the same age. *p < 0.05, **p < 0.01, ***p < 0.001, ****p < 0.0001. SC: status convulsion. NC negative control adenovirus vector carrying a nonsense gene sequence, AV adenovirus vectors used to specifically silence the AMIGO3 gene, OAV adenovirus vector for AMIGO3 gene overexpression.

Expression of ROCK/RhoA and PI3K/AKT signaling pathway proteins in the cortex was detected by ELISA (Figure 8). Compared with the normal group, p−AKT and AKT expression levels decreased significantly at days 5 to 20 after SC (*P* < 0.01) (Figures 8A,B). Compared with the SC group, the expression levels of p−AKT and AKT in the AV group were higher at day 20 (*P* < 0.05) (Figures 8A,B). The ratio of p−AKT/AKT showed no statistical significance among groups at different timepoints (Figure 8C). The expression of p−RhoA increased significantly from days 1 to 20 after SC (*P* < 0.05) (Figure 8D). Compared with the SC group, the expression of p−RhoA in the OAV group was higher than at day 5, while the level in the AV group was lower at days 1 and 20 (*P* < 0.05) (Figure 8D). The expression of RhoA increased significantly at days 5 and 20 after SC (*P* < 0.05) (Figure 8E). Compared with the SC group, the expression of RhoA in the OAV group was higher at day 1, while that in the AV group was lower at days 5 and 20 after SC (*P* < 0.05) (Figure 8E). The ratio of p−RhoA/RhoA at day 20 showed statistical significance in the AV group compared with SC group (Figure 8F).

## Discussion

The distribution of AMIGO3 in the hippocampus was found to be consistent with that of Lingo-1. We also found that AMIGO3 protein levels showed a similar pattern of increasing expression as that observed for Lingo-1, with peak expression at day 20 after SC. However, in the mouse cortex, AMIGO3 expression was significantly higher than Lingo-1 expression from 1 to 20 days after SC, supporting the hypothesis that AMIGO3 plays an important role in the formation of abnormal neural circuits in the immature brain after SC. In this context, AMIGO3-targeted therapies may provide more rapid benefits than those focused on LINGO1 in terms of promoting oligodendrocyte precursor cell (OPC) differentiation and remyelination during the process of epileptogenesis (Mi et al., 2007; Ahmed et al., 2013; Foale et al., 2017).

In this study, we found that myelin sheath integrity in immature mice cortex was damaged during the process of epileptogenesis induced by kainic acid. Inhibition of AMIGO3 expression alleviated the damage to myelin sheaths induced by SC, and increased the levels of MBP in myelinating oligodendrocytes. The opposite effect on the myelin sheath damage caused by SC was observed after AMIGO3 overexpression. These results suggest that AMIGO3 signaling is involved in the pathophysiological mechanism of seizure-induced myelin damage. The mechanism by which AMIGO3 inhibits OPC maturation and myelin production after SC remains unclear, although it has been speculated that AMIGO3 and Lingo-1 share the same molecular mechanisms in these processes. Lingo-1 has been shown to inhibit the activation of the intracellular PI3K (Inoue et al., 2007), thus preventing activation of the AKT signaling pathway (Sun et al., 2015). Our results showed that the PI3K/AKT signaling pathway has no significant change after regulation of AMIGO3, suggesting that other possible mechanisms may be involved in this pathological process.

Studies have confirmed that axon sprouting is modulated by three major myelin-derived inhibitors (NogoA, OMgp, and MAG) (Cafferty et al., 2010; Lee et al., 2010). The extracellular signals generated by binding of these inhibitors of the Nogo receptor (NgR) are transmitted to AMIGO3 via p75 or TNF receptor orphan Y (TROY) to activate downstream signals, such as ROCK/RhoA signaling pathway, that mediate axonal outgrowth and regeneration (Ahmed et al., 2013). Our data showed the levels of MAG increased at 1 and 5 days after SC, and the levels of MOG significantly increased at 20 days after SC after AMIGO3 overexpression. Suppressing AMIGO3 expression inhibited ROCK/RhoA signaling pathway at 20 days after SC. AMIGO3 expression increased rapidly after axotomy of both retinal ganglion cells and dorsal root ganglion neurons (DRGN) (Ahmed et al., 2013). A reduction in AMIGO3 levels in these models is associated with dorsal column and optic nerve axon regeneration, while AMIGO3 antagonism enhanced axon outgrowth of DRGN in the presence of inhibitory myelin extracts. Our results confirm that, similar to Lingo-1, AMIGO3 inhibits axon outgrowth and regeneration. It can be speculated that AMIGO3 can replace Lingo-1 in the NgR1 receptor complex to mediate myelin-induced inhibition of axon outgrowth during epileptogenesis. Moreover, AMIGO3 was found to be upregulated more rapidly (within 24 h) than Lingo-1 after spinal cord injury, while the levels of Lingo-1 mRNA remained unchanged at day 10 (Ahmed et al., 2013). Thus, AMIGO3 antagonism is implicated as a potential therapeutic strategy for alleviating the inhibition of neurite regeneration and preventing growth cone collapse.

Changes in synaptic plasticity increase susceptibility to seizures after SC. Normal numbers and morphology of synapses are the cornerstones of synaptic plasticity. The active functional region of the presynaptic membrane, known as the AZ, is anchored by synaptic vesicles containing neurotransmitters. The length of AZ corresponds to the area of contact with synapses and the ability of the presynaptic membrane to reabsorb neurotransmitters in the synaptic cleft (Rosenmund et al., 2003; Szule et al., 2015). PSD was defined to be a cytoskeletal specialized region of the postsynaptic membrane contained a variety of functional proteins to conduct neural signals, which is characterized by high-density electron thickness observed under TEM. In our study, the numbers of synapses decreased at days 1 and 5 after SC, indicating synaptic plasticity was disrupted by seizures. Furthermore, following inhibition of AMIGO3 expression, the number of synapses was increased at days 1 and 5 after SC. The length of the AZ decreased at days 1 and 20 and the thickness of PSD increased at days 5 and 20 after SC. These results suggest that the structural changes in the synapse following SC can be alleviated by inhibiting AMIGO3 expression. However, the underlying mechanisms remain to be elucidated.

Our study confirmed that AMIGO3 is highly expressed in the cortex in an animal model of SC, with expression maintained to 20 days after SC in a pattern of changes that are similar to those observed for Lingo-1. SC disrupts the integrity of myelin sheaths, axon growth and synaptic plasticity in immature brains. We also showed that silencing of AMIGO3 expression alleviated the damage to myelinated fibers and synapses after SC. In contrast, upregulation of AMIGO3 expression inhibited the axon growth induced by SC at 20 days after SC. Antagonizing AMIGO3 rather than Lingo-1 is likely to be a more effective therapeutic strategy to protecting the formation of abnormal neural circuits after SC. Due to the similar protective effect of Lingo-1 and AMIGO3, a combinational treatment of inhibiting both may exert synergistic effect to alleviate convulsion-induced brain injury. Furthermore, after downregulation of AMIGO3 expression, the ROCK/RhoA signaling pathway was inhibited at 20 days after SC. Thus, our findings indicate that the effects of AMIGO3 on neural circuits are mediated via the ROCK/RhoA signaling pathways.

Thus, compared to Lingo-1, our study highlights the potential of AMIGO3 as a more effective therapeutic target to decrease abnormal neural circuit formation in the immature brain after SC and demyelinating diseases, although further studies are required to fully elucidate the underlying mechanism.

## Data Availability Statement

The raw data supporting the conclusions of this article will be made available by the authors, without undue reservation.

## Ethics Statement

The animal study was reviewed and approved by the Laboratory Animal Care of Chongqing Medical University.

## Author Contributions

XS and LJ conceived and designed the experiments. XL contributed to data acquisition and manuscript writing. YP, JG, ZF, DH, HL, LC, and HC contributed reagents, materials, and analytical tools. All authors read and approved the manuscript.

## Conflict of Interest

The authors declare that the research was conducted in the absence of any commercial or financial relationships that could be construed as a potential conflict of interest.

## Publisher’s Note

All claims expressed in this article are solely those of the authors and do not necessarily represent those of their affiliated organizations, or those of the publisher, the editors and the reviewers. Any product that may be evaluated in this article, or claim that may be made by its manufacturer, is not guaranteed or endorsed by the publisher.

## References

[B1] Ahmed Z. Dent R. G. Suggate E. L. Barrett L. B. Seabright R. J. Berry M. (2005). Disinhibition of neurotrophin-induced dorsal root ganglion cell neurite outgrowth on CNS myelin by siRNA-mediated knockdown of NgR, p75NTR and Rho-A. *Mol. Cell. Neurosci.* 28 509–523. 10.1016/j.mcn.2004.11.002 15737741

[B2] Ahmed Z. Douglas M. R. John G. Berry M. Logan A. (2013). AMIGO3 is an NgR1/p75 co-receptor signalling axon growth inhibition in the acute phase of adult central nervous system injury. *PLoS One* 8:e61878. 10.1371/journal.pone.0061878 23613963 PMC3628841

[B3] Aktas O. Albrecht P. Hartung H. P. (2016). Optic neuritis as a phase 2 paradigm for neuroprotection therapies of multiple sclerosis: update on current trials and perspectives. *Curr. Opin. Neurol.* 29 199–204. 10.1097/wco.0000000000000327 27035900

[B4] Aldenkamp A. P. Baker G. A. Meador K. J. (2004). The neuropsychology of epilepsy: what are the factors involved? *Epilepsy Behav.* 5 S1–S2. 14725840 10.1016/j.yebeh.2003.11.001

[B5] Andrews J. L. Fernandez-Enright F. (2015). A decade from discovery to therapy: lingo-1, the dark horse in neurological and psychiatric disorders. *Neurosci. Biobehav. Rev.* 56 97–114. 10.1016/j.neubiorev.2015.06.009 26143511

[B6] Cafferty W. B. Duffy P. Huebner E. Strittmatter S. M. (2010). MAG and OMgp synergize with Nogo-A to restrict axonal growth and neurological recovery after spinal cord trauma. *J. Neurosci.* 30 6825–6837. 20484625 10.1523/JNEUROSCI.6239-09.2010 PMC2883258

[B7] Chen Y. Aulia S. Li L. Tang B. L. (2006). AMIGO and friends: an emerging family of brain-enriched, neuronal growth modulating, type I transmembrane proteins with leucine-rich repeats (LRR) and cell adhesion molecule motifs. *Brain Res. Rev.* 51 265–274. 10.1016/j.brainresrev.2005.11.005 16414120

[B8] Foale S. Berry M. Logan A. Fulton D. Ahmed Z. (2017). LINGO-1 and AMIGO3, potential therapeutic targets for neurological and dysmyelinating disorders? *Neural Regen. Res.* 12 1247–1251. 10.4103/1673-5374.213538 28966634 PMC5607814

[B9] He R. Han W. Song X. Tang X. Cheng L. Jiang L. (2017). Effect of fasudil on cognitive function following status convulsion in rats. *Mol. Med. Rep.* 16 119–126. 28534935 10.3892/mmr.2017.6615 PMC5482154

[B10] Hermann B. Seidenberg M. Lee E. J. Chan F. Rutecki P. (2007). Cognitive phenotypes in temporal lobe epilepsy. *J. Int. Neuropsychol. Soc.* 13 12–20. 17166299 10.1017/S135561770707004X

[B11] Inoue H. Lin L. Lee X. Shao Z. Mendes S. Snodgrass-Belt P. (2007). Inhibition of the leucine-rich repeat protein LINGO-1 enhances survival, structure, and function of dopaminergic neurons in Parkinson’s disease models. *Proc. Natl. Acad. Sci. U. S. A.* 104 14430–14435. 10.1073/pnas.0700901104 17726113 PMC1955463

[B12] Jepson S. Vought B. Gross C. H. Gan L. Austen D. Frantz J. D. (2012). LINGO-1, a transmembrane signaling protein, inhibits oligodendrocyte differentiation and myelination through intercellular self-interactions. *J. Biol. Chem.* 287 22184–22195. 22514275 10.1074/jbc.M112.366179 PMC3381180

[B13] Kuja-Panula J. Kiiltomäki M. Yamashiro T. Rouhiainen A. Rauvala H. (2003). AMIGO, a transmembrane protein implicated in axon tract development, defines a novel protein family with leucine-rich repeats. *J. Cell Biol.* 160 963–973. 10.1083/jcb.200209074 12629050 PMC2173769

[B14] Ledford H. (2015). Drug that boosts nerve signals offers hope for multiple sclerosis. *Nature* 520:417. 10.1038/520417a 25903604

[B15] Lee J. K. Geoffroy C. G. Chan A. F. Tolentino K. E. Crawford M. J. Leal M. A. (2010). Assessing spinal axon regeneration and sprouting in Nogo-, MAG-, and OMgp-deficient mice. *Neuron* 66 663–670. 10.1016/j.neuron.2010.05.002 20547125 PMC2896331

[B16] Mi S. Hu B. Hahm K. Luo Y. Kam Hui E. S. Yuan Q. (2007). LINGO-1 antagonist promotes spinal cord remyelination and axonal integrity in MOG-induced experimental autoimmune encephalomyelitis. *Nat. Med.* 13 1228–1233. 17906634 10.1038/nm1664

[B17] Mi S. Lee X. Shao Z. Thill G. Ji B. Relton J. (2004). LINGO-1 is a component of the Nogo-66 receptor/p75 signaling complex. *Nat. Neurosci.* 7 221–228. 10.1038/nn1188 14966521

[B18] Reinholdson J. Olsson I. Edelvik A. Hallböök T. Lundgren J. Rydenhag B. (2015). Long-term follow-up after epilepsy surgery in infancy and early childhood–a prospective population based observational study. *Seizure* 30 83–89. 26216690 10.1016/j.seizure.2015.05.019

[B19] Rosenmund C. Rettig J. Brose N. (2003). Molecular mechanisms of active zone function. *Curr. Opin. Neurobiol.* 13 509–519. 10.1016/j.conb.2003.09.011 14630212

[B20] Schraegle W. A. Titus J. B. (2017). The relationship of seizure focus with depression, anxiety, and health-related quality of life in children and adolescents with epilepsy. *Epilepsy Behav.* 68 115–122. 10.1016/j.yebeh.2016.12.009 28142130

[B21] Song X. J. Han W. He R. Li T. Y. Xie L. L. Cheng L. (2018). Alterations of Hippocampal Myelin Sheath and Axon Sprouting by Status Convulsion and Regulating Lingo-1 Expression with RNA Interference in Immature and Adult Rats. *Neurochem. Res.* 43 721–735. 10.1007/s11064-018-2474-2 29383653

[B22] Sun J. J. Ren Q. G. Xu L. Zhang Z. J. (2015). LINGO-1 antibody ameliorates myelin impairment and spatial memory deficits in experimental autoimmune encephalomyelitis mice. *Sci. Rep.* 5:14235. 10.1038/srep14235 PMC4585639 26383267

[B23] Szule J. A. Jung J. H. McMahan U. J. (2015). The structure and function of ‘active zone material’ at synapses. *Philos. Trans. R. Soc. Lond. B Biol. Sci.* 370:20140189. 10.1098/rstb.2014.0189 26009768 PMC4455758

[B24] Ye Y. Xiong J. Hu J. Kong M. Cheng L. Chen H. (2013). Altered hippocampal myelinated fiber integrity in a lithium-pilocarpine model of temporal lobe epilepsy: a histopathological and stereological investigation. *Brain Res.* 1522 76–87. 10.1016/j.brainres.2013.05.026 23727401

